# Dr. Addison

**Published:** 1860-09

**Authors:** 


Dr. Addison.—The late distinguished physician of Guy's Hospital, since last Christmas had been in bad health, and only recently he suffered from an attack of gall-stones. A trial in which he was engaged, and the death of his friend, the late Dr. Todd, so harassed him, that he fell into a state of melancholy, and on Friday, the twenty-ninth of June, he threw himself from a height at Brighton, and was instantly killed. At the time of his decease, Dr. Addison was about seventy years of age. He took his degree in 1815, and, removing subsequently to London, he received the appointment of House Surgeon to the Lock Hospital. In

1824, he was appointed Assistant Physician to Guy's Hospital, and, in opposition to the opinions of some of his confreres, he practiced and appreciated the principles of Laennec, and soon, by the skillful use of the stethoscope, became a proficient in the diagnosis of affections of the chest. In 1827, he began a course of lectures on Materia Medica, and probably received larger fees for his course than any other private teacher at that time in London. Ten years later he was appointed physician to the hospital, and, joining Dr. Bright in the Chair of Medicine, commenced conjointly with him the publication of a work on "Medicine," but one volume of which ever appeared, and the greater portion of that emanated from the pen of Dr. Addison.

Dr. Addison was particularly celeorated as a diagnostician, and although hardly belonging to his branch, he was considered authority upon the subject of syphilis. As a pupil of the distinguished Bateman, he soon became thoroughly acquainted with skin affections; and the beautiful wax models in the museum of Guy's Hospital certainly denote that he was an accomplished dermatologist. He was, moreover, a good pathologist, being in daily attendance at the dead-room up to two years before his death. As a writer he cannot be considered copious. His medical contributions are to be found mainly in Guy's Hospital Reports, and the most important of these pertain to pneumonia, phthisis, the cerebral complications of Bright's disease, and the fatty liver.

Dr. Addison's name will be perpetuated more particularly on account of a disease which bears his name, but which he termed melasma supra-renale. But few years have elapsed since he pointed out the connection between bronzed skin and disease of the supra-renal capsules, and we are glad to know that to this peculiar affection is given the name of morbus Addisonii.

